# Supplementary material for: De novo annotation of lncRNA HOTAIR transcripts by long-read RNA capture-seq reveals a differentiation-driven isoform switch
Source: BMC Genomics. 2022 Sep 17;23:658. doi: 10.1186/s12864-022-08887-w (PMC9482196; doi:10.1186/s12864-022-08887-w)
Supplement: Supplementary file 1 — Additional file 1: Figure S1. Validation of adipogenic differentiation efficiency. Figure S2. Genome browser view of reference HOTAIR transcripts for SQANTI characterization. Figure S3. Semi-quantitative RT-PCR replicates. [file 12864_2022_8887_MOESM1_ESM.docx]

**Additional file 1**

**
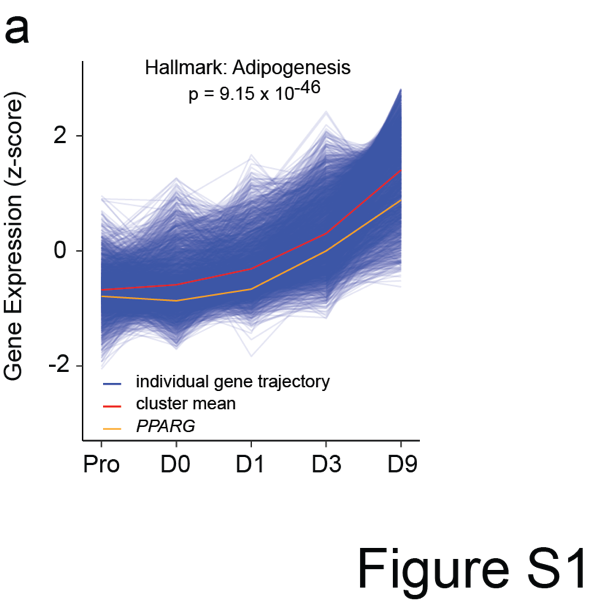
**

**Figure S1**. **Validation of adipogenic differentiation efficiency. a** Cluster of differentially expressed genes induced over the adipogenic RNA-seq time-course (n=3, adjusted p-value).


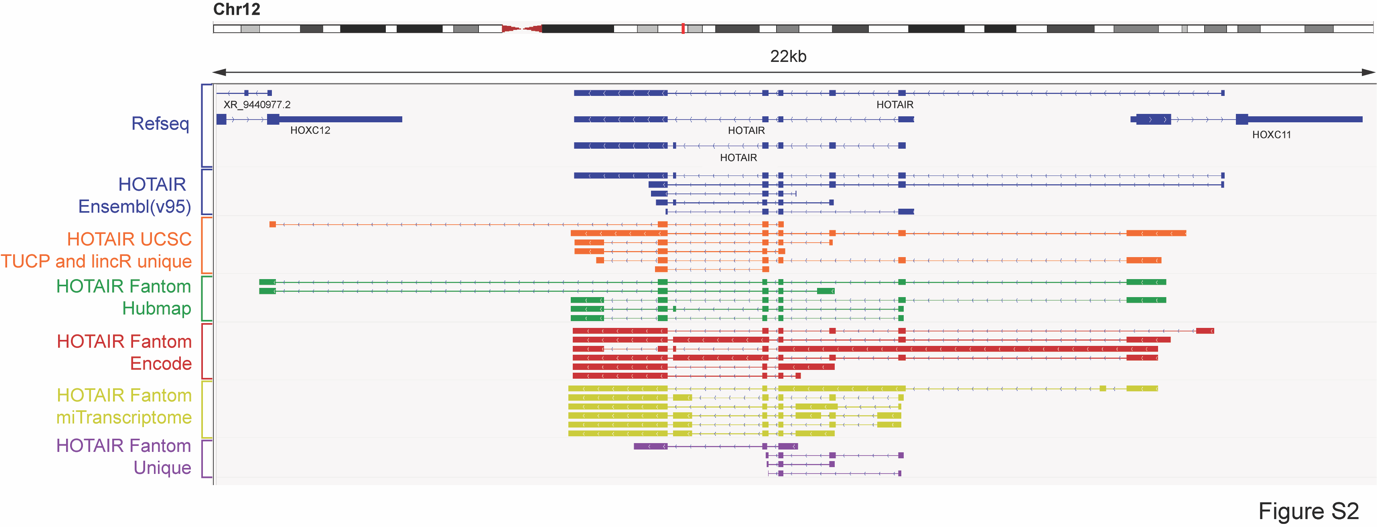
**Figure S2.** Genome browser view of reference *HOTAIR* transcripts for SQANTI characterization


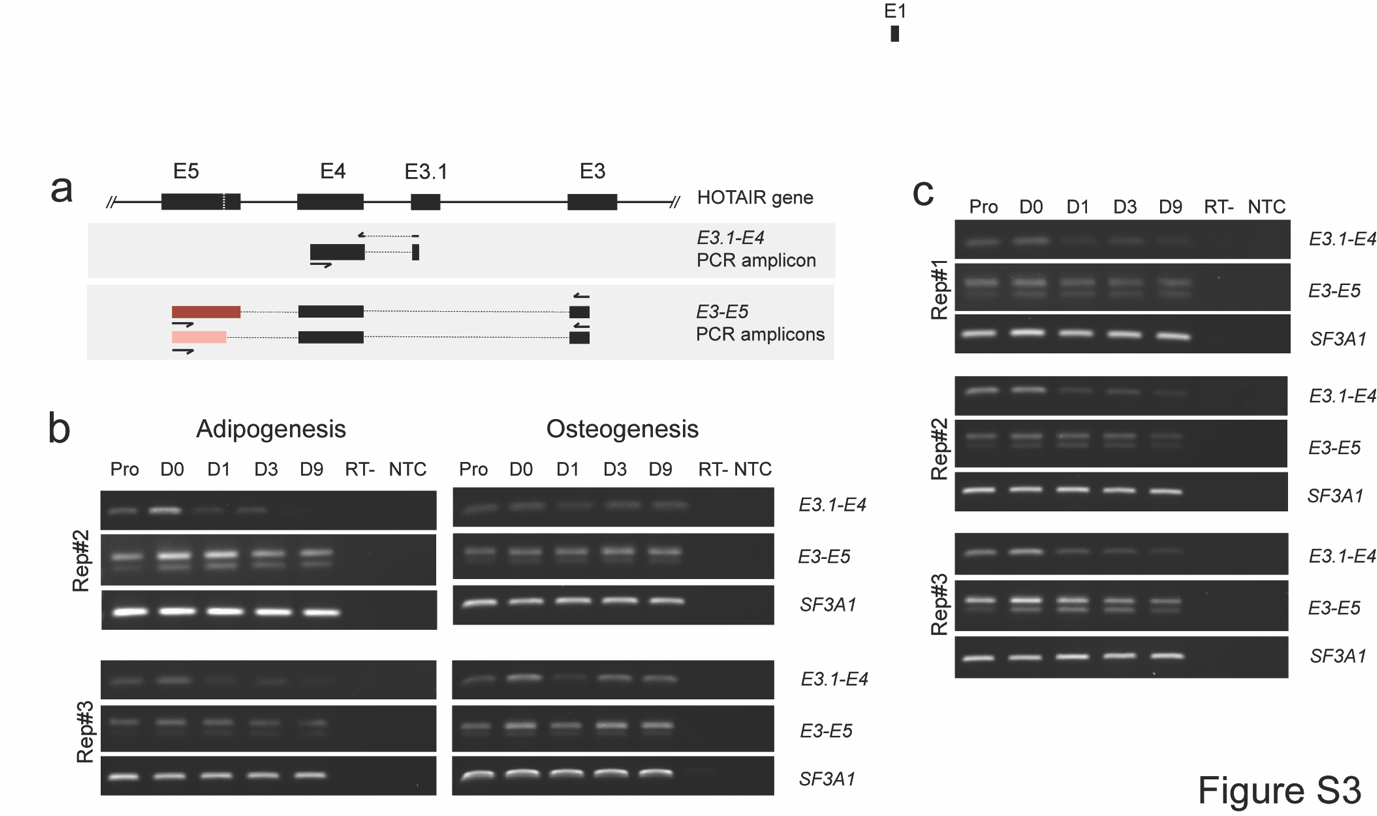


**Figure S3. Semi-quantitative RT-PCR replicates. a** Schematic representation of *HOTAIR* PCR amplicons using primer pairs located in exons E3.1 and E4 or E3 and E5. **b** Semi-quantitative RT-PCR analysis of *HOTAIR* isoform expression during adipogenic and osteogenic differentiation using primers located in *HOTAIR* exons E3.1-E4 and E3-E5 in ASCs from donor 1 (replicates #2 and #3; Full-length gels are presented in **Additional file 4 Fig. S4** and **S6**) and **c** donor 2 (Full-length gels are presented in **Additional file 4 Fig. S5**) RT-: no reverse transcriptase control; NTC: no template control. SF3A1 is shown as a loading control.
