# Supplementary material for: De novo annotation of lncRNA HOTAIR transcripts by long-read RNA capture-seq reveals a differentiation-driven isoform switch
Source: BMC Genomics. 2022 Sep 17;23:658. doi: 10.1186/s12864-022-08887-w (PMC9482196; doi:10.1186/s12864-022-08887-w)
Supplement: Supplementary file 4 — Additional file 4: Figure S4. Uncropped gels for Fig.3f and Additional file 1, FigS3b left panel. Figure S5. Uncropped gels for Additional file 1, Fig. S3c. Figure S6. Uncropped gels for Fig.4g and Additional file 1, Fig. S3b right panel. Figure S7. Uncropped gels for Fig.5b. [file 12864_2022_8887_MOESM4_ESM.docx]

**Additional file 4 (pdf)**

**
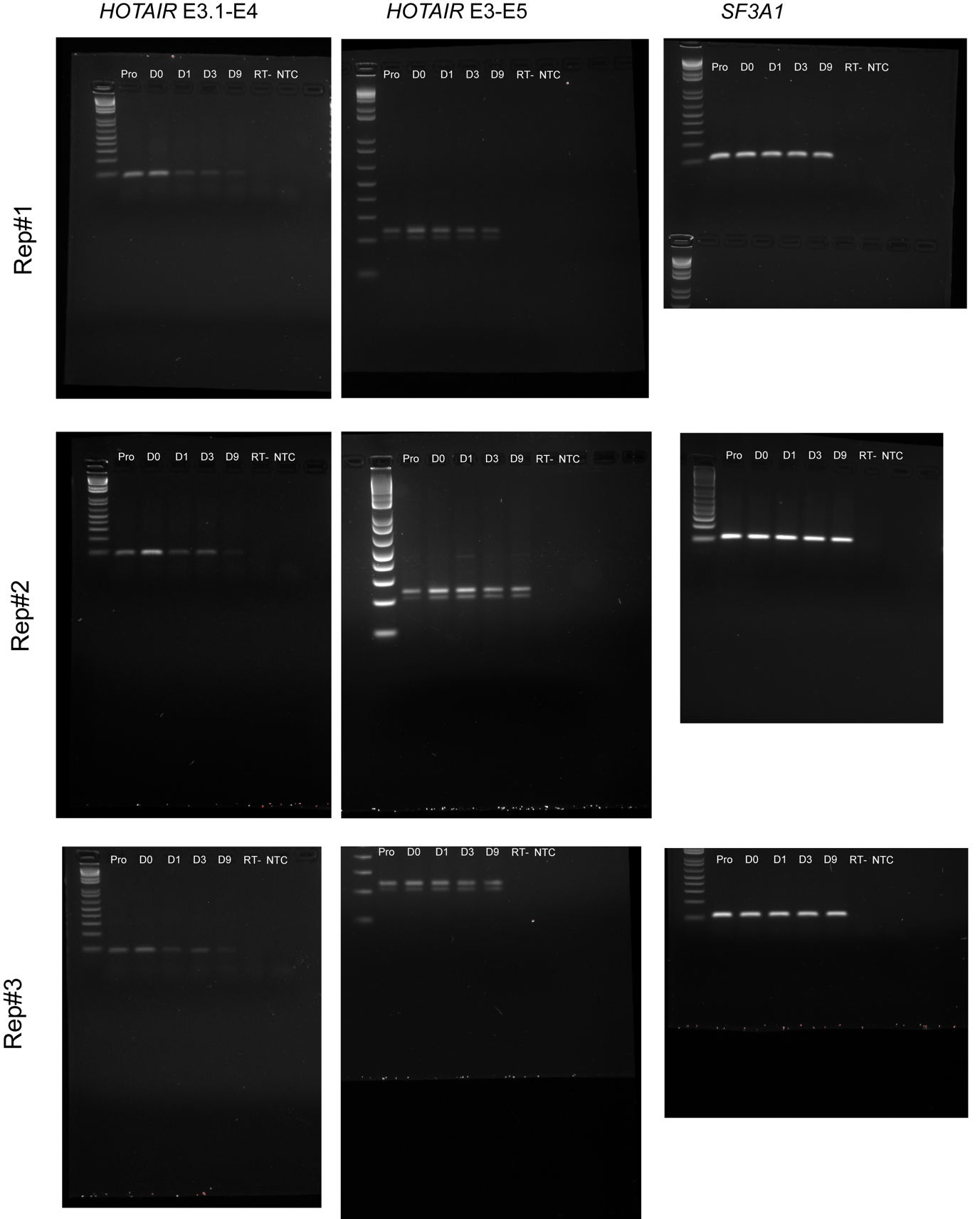
**

**Figure S4.** Uncropped gels for **Fig.3f** and **Additional file 1, FigS3b** left panel

**
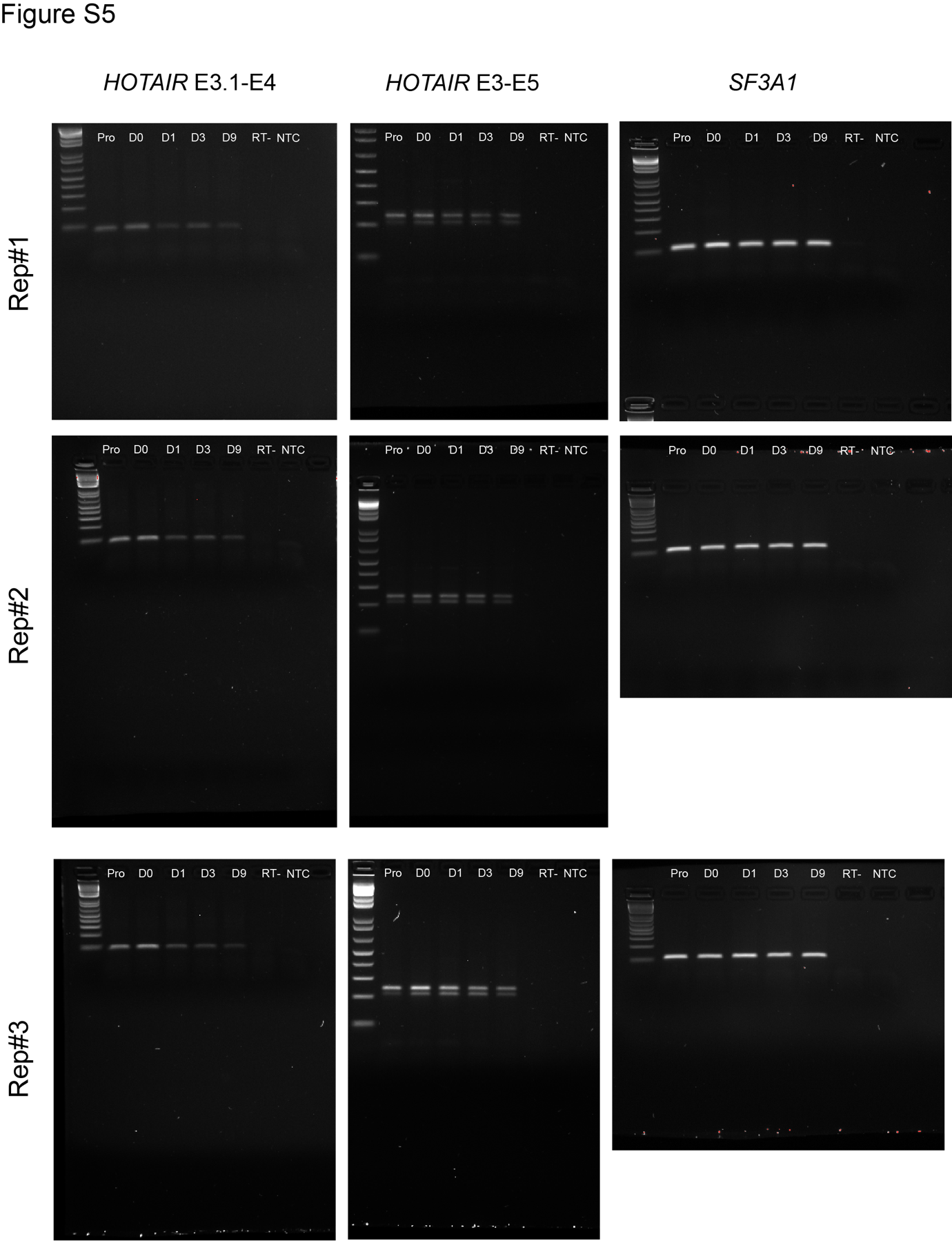
Figure S5.** Uncropped gels for **Additional file 1, Fig. S3c**

**
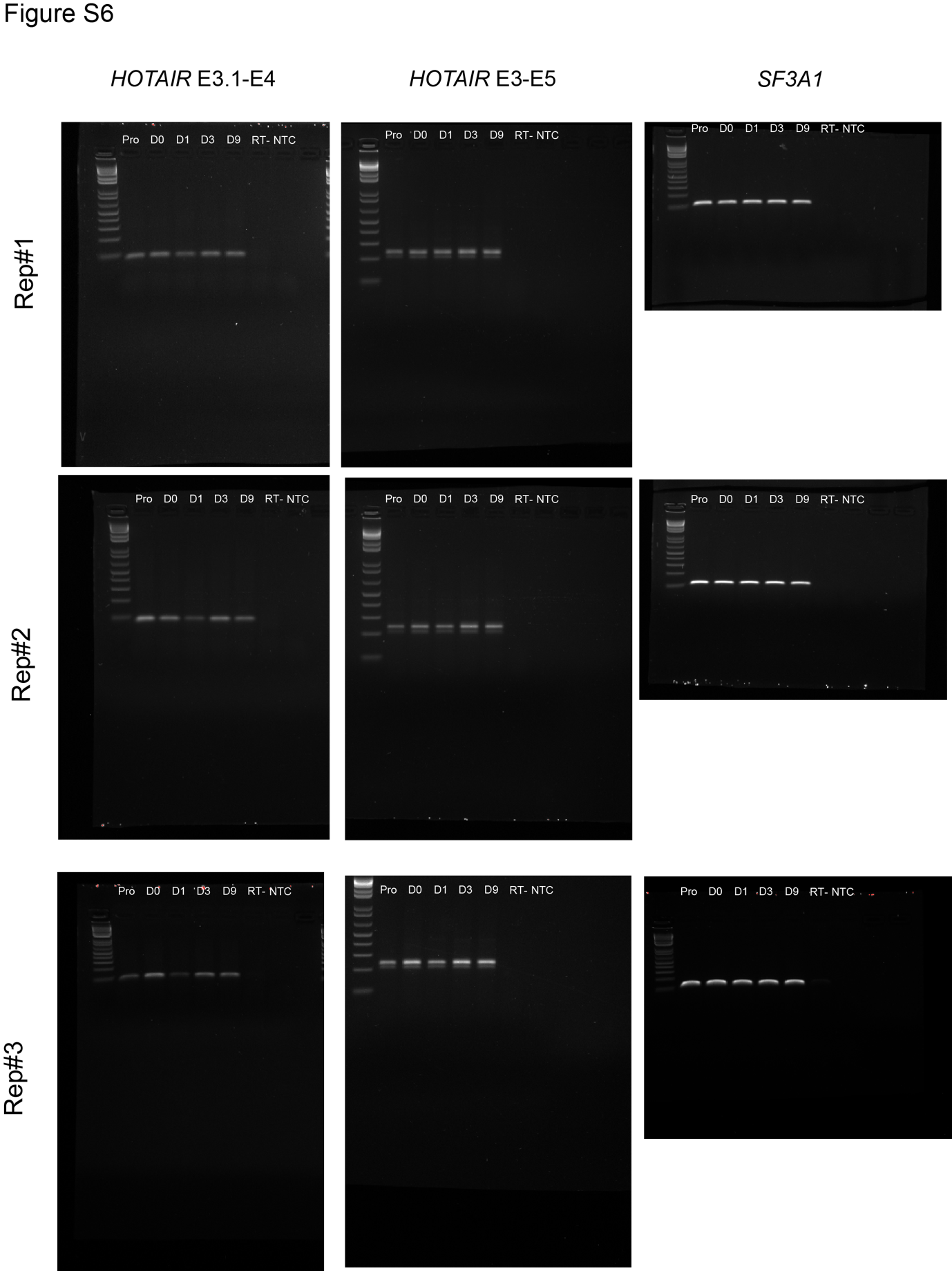
**

**Figure S6.** Uncropped gels for **Fig.4g** and **Additional file 1, Fig. S3b** right panel

**
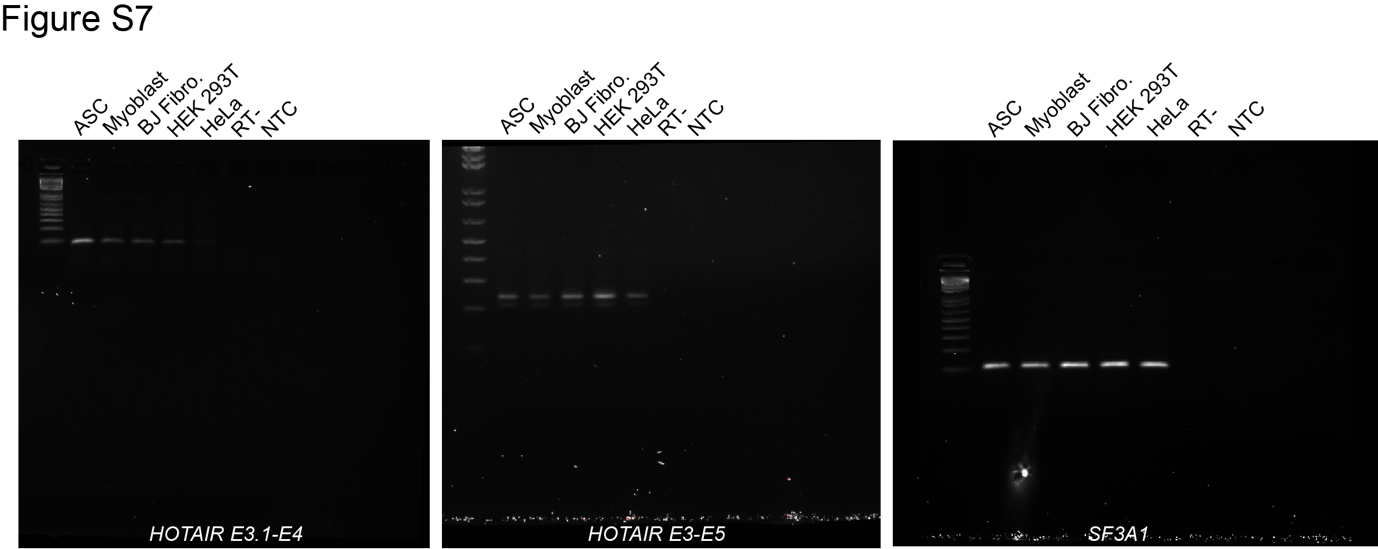
**

**Figure S7.** Uncropped gels for **Fig.5b**
